# Compassion fatigue and substance use among nurses

**DOI:** 10.1186/s12991-018-0183-5

**Published:** 2018-03-13

**Authors:** Reem Jarrad, Sawsan Hammad, Tagreed Shawashi, Naser Mahmoud

**Affiliations:** 1 Clinical Nursing Department-School of Nursing, The University of Jordan, Amman, 11942 Jordan; 2 Community Health Department-School of Nursing, The University of Jordan, Amman, Jordan; 3 Jordan Ministry of Health-Fuheis Psychiatric Hospital, Amman, Jordan

**Keywords:** Compassion fatigue, Substance use, Nurses, Demographic variables

## Abstract

**Aim:**

This study aimed to detect if there were differences in compassion fatigue (CF) among nurses based on substance use and demographic variables of gender, marital status, type of health institution and income.

**Background:**

Compassion fatigue is considered an outcome of poorly handled stressful situations in which nurses may respond with self-harming behaviours like substance use. Evidence in this area is critically lacking.

**Methods:**

This study used a descriptive design to survey differences in CF of 282 nurses. The participants completed a demographic survey and indicated whether they consume any of the following substances on a frequent basis: cigarettes, sleeping pills, power drinks, anti-depressant drugs, anti-anxiety drugs, coffee, analgesics, amphetamines and alcohol. Compassion Fatigue scores were surveyed using CF self-test 66 items developed by Stamm and Figely (Compassion satisfaction and fatigue test. http://www.isu.edu/~bhstamm/tests.htm, 1996).

**Results:**

There were significant differences in CF scores in favour of nurses who used cigarettes, sleeping pills, power drinks, anti-depressants and anti-anxiety drugs. While no significant differences in CF were found between nurses who used coffee, analgesics, amphetamines and alcohol, significant differences in nurses’ CF were found in relation to type of institution, gender and marital status. But nurses’ income did not bring differences to CF scores.

**Conclusion:**

Nurses who might be lacking resilience cope negatively with CF using maladaptive negative behaviours such as substance use.

**Implications for nursing management:**

Nursing management should be aware of the substance use drive among nurses and build organizational solutions to overcome compassion fatigue and potential substance use problems.

## Introduction

Compassion fatigue (CF) is a recent concept that refers to the emotional and physical exhaustion that affects helping professionals and caregivers over time. It is associated with a gradual desensitization to patient stories, a decrease in quality of care, an increase in clinical errors, higher rates of depression and anxiety disorders and rising rates of stress leave and a sense of humiliation in workplace climate [1].

Compassion fatigue in nurses can be explained as a cumulative and progressive absorption process of patient’s pain and suffering formed from the caring interactions with patients and their families. The physical, emotional, spiritual, social and organizational consequences of CF are so extensive that they threaten the existential integrity of the nurse [2]. Such consequences include, but not limited to, decreased level of job satisfaction, decreased productivity, increased rates of absenteeism, burnout, turnover, stress, insomnia, nightmares, headaches, gastrointestinal complaints, anxiety and depression [3].

Compassion fatigue can happen to any nurse, at any time during the job course, though some nurses may be at greater risk to develop CF than other nurses. For example, those who work in oncology, emergency, intensive care units, paediatric units and hospice care are at a greater risk of developing CF because of the frequent encounters with patient/family tragedies and deaths [4].When close, caring relationships are formed with patients, the risk of CF is increased. Sometimes a particular patient may remind the nurse of someone important in his or her life. If that patient dies, the nurse may be triggered emotionally in the most debilitating ways [5]. One of the greatest risks for compassion fatigue comes when nurses forgo their own self-care while immersing themselves intensely in their patients’ traumatization, suffering, grief and pain [6].

## Theoretical framework

Nurses are particularly vulnerable to traumatic experiences and resultant CF because they usually enter the lives of patients at very critical health junctures and become directly and deeply involved in providing multidimensional care as well as end-of-life care [7]. The negative effects of providing care are aggravated by the severity of the traumatic experiences to which nurses are exposed. Those traumatic experiences may bring to life a group of unpleasant feelings such as exhaustion, anger, irritability, diminished sense of enjoyment and impaired ability to make decisions and care for patients. Subsequently, some nurses develop negative coping behaviours including alcohol and drug use or abuse [8, 9].

There are several styles with which a care giver respond to CF poorly handled stressful situations. For example, the Coping Inventory for Stressful Situations, by Endler and Parker [10] identified three coping styles: task-oriented coping (i.e. taking actions to solve the situation), emotion-oriented coping (e.g. self-blame and anxiety), and avoidance-oriented coping (replacement behaviours to substitute the problem); the last two coping styles may result in care giver self-harm and self-destructive behaviours that include, but are not limited to, substance use. In support of this explanative framework Adriaenssens et al. [11] asserted that what matters is how individuals respond to stressors not the stressors themselves. The response could be in an action oriented and problem solving manner (adaptive coping response) or resort to ineffective coping responses and defensive mechanisms like substance use and withdrawal.

An understanding of the coping responses of nurses can help develop resilience-promoting interventions tailored to ease the resolution of CF issues and maximize retention rates in work places [12]. Those outcomes are directly supported by the concept of resilience which implies the ability to effectively cope and adapt when faced with loss or hardship and minimize the negative results of exposure to adverse situations [13].

Zander et al. [14] aimed to develop strategies that can be implemented at an organizational level to support the development of resilience in nurses and hence counteract the wide spectrum of negative consequences of compassion fatigue. Ginzburg [15] stated that resilience is a survival skill that is often manifested as the difference between individuals’ conceptualizing themselves as survivors versus victims and individuals who can take care of themselves and others, versus those who become unable to care for themselves or others when subjected to significant stressors; which is definitely an unwanted outcome in health care facilities.

Hereby, this study hypothesized that nurses with the highest level of compassion fatigue may turn to maladaptive, less resilient, negative coping behaviours to manage their feelings. Such behaviours may include surrendering to some forms of substance use or abuse.

## Research questions

This study aimed to detect if there were differences in CF scores, the dependent variable, among Jordanian nurses based on substance use. The independent variables were: cigarette smoking, sleeping pills, power drinks, anti-depressant drugs, anti-anxiety drugs, coffee, analgesics, amphetamines and alcohol. The second objective was to evaluate if there were differences in CF scores among Jordanian nurses based on demographic variables of: gender, type of health institution, marital status and income.

## Methods

### Sample and design

This study used a descriptive cross-sectional convenient sampling design to survey CF and some associated demographic and drug use related variables among Jordanian nurses. The sample included 282 nurses selected from three types of major and high occupancy rate hospitals covering psychiatric, governmental and educational sectors. The sample included nurses who had spent at least 3 months in the current unit.

The areas selected from within the governmental and educational hospitals included several types of intensive care units (ICUs), emergency departments, medical and surgical floors which received some oncology cases that clinically ranged from early diagnosis to terminal illness conditions. The specialized psychiatric hospital had clients with a variety of mental illnesses such as schizophrenia, depression and bipolar disorders in variable levels of acuity.

## Measures

The participants were asked to complete a single page demographic form which had two sections. Section one included questions regarding: age, income, duration of experience, gender, unit, hospital type, marital status and income, while section two requested a yes/no response to statements about frequent use of cigarettes, sleeping pills, power drinks, anti-depressant drugs, anti-anxiety drugs, coffee, analgesics, amphetamines and alcohol.

The Compassion Fatigue scores were surveyed using a face and content validated translation to Arabic version of the CF self-test which was adapted with permission of Dr. Charles Figely [17]. This survey measures three concepts which are: compassion fatigue, compassion satisfaction and burn out. The survey has been used by many researchers in variable target groups such as: educators, clinicians, social workers, nurses, therapists, chaplains, counsellors, etc. The compassion fatigue section of the test showed a strong alpha value of 0.87; the analysis of variance did not provide evidence of differences based on country of origin, type of work or sex when age was used as a control variable [16].

The CF self-test has 66 items (Appendix 1). Each item is evaluated by the care provider on a Likert scale out of five. Zero means never; one means rarely, two means a few times, three means somewhat often, four means often and five means very often. The CF score is the sum of the following 23 test items: 4, 6, 7, 8, 12, 13, 15, 16, 18, 20–22, 28, 29, 31–34, 36, 38–40 and 44. If the calculated score is 26 or less there would be an extremely low risk for CF. When the score is 27–30 there would be low risk of CF. Score a 31–35 refers to moderate CF risk. Whereas a score of 36–40 is considered a high CF risk and a score of 41 or above indicates an extremely high risk for CF [17].

## Results and statistical analysis

### Sample characteristics

The sample of this study consisted of 64% (*n* = 181) females and 36% (*n* = 101) males. Their mean age was 32.3 years (SD = 6.6). Half the sample were working in governmental hospitals (*n* = 141), the minority were in educational (training) hospitals 17% (*n* = 48) and almost a third were from a specialized psychiatric institution (*n *= 93). The majority of the participants were married 68% (*n* = 98) and 74% (*n* = 209) have a monthly individual income of 600 JD or less. The participants had mean of 9.9 years of experience as nurses and 6.3 years of experience in the current unit or ward. This is quite enough range of time to measure certain nurses’ emotional outcomes such as compassion fatigue (Table 1).

**Table 1:** Description of the personal demographics, *N* = 282

Variable	*n* (%)	*M* (SD)
Type of hospital		
Psychiatric hospital	93 (33)	
Governmental hospital	141 (50)	
Educational (training) hospital	48 (17)	
Gender		
Male	101 (36)	
Female	181 (64)	
Age		32.3 (6.6)
Marital status		
Married	192 (68)	
Not married	90 (32)	
Experience as a nurse		9.9 (6.5)
Experience in the current unit/ward		6.3 (5.4)
Income		
Less or equal 600	209 (74)	
More than 600	73 (26)	

### Substance use among Jordanian nurses

Table 2 displays the frequency of substance use among the nurses participating in the study. The highest frequencies were for coffee 69% (*n* = 194), analgesic drugs 41% (*n* = 115), cigarette smoking 29% (*n* = 81), and power drinks 52 (18%). The lowest frequencies were for alcohol 8% (*n* = 23), amphetamines 12% (*n* = 33), antidepressants 15% (*n* = 41), sleeping pills 16% (46), and anti-anxiety drugs 17% (*n* = 48).

**Table 2:** Description of the substance use among nurses, *N* = 282

Variable	Yes *n* (%)	No *n* (%)
Smoking	81 (29)	201 (71)
Alcohol	23 (8)	259 (92)
Sleeping pills	46 (16)	236 (84)
Power drinks	52 (18.0)	230 (82)
Coffee	194 (69)	88 (31)
Antidepressants	41 (15)	241 (86)
Anti-anxiety drugs	48 (17)	234 (83)
Stimulants (amphetamines)	33 (12)	249 (88)
Analgesic drugs	115 (41)	167 (59)

### Differences in compassion fatigue level in relation to socio-demographic and substance use variables

In this study, the mean as well as the median scores of CF among all nurses was 41 (SD = 17.7). This aligns with the category of “extremely high risk for CF”, regardless of any contributing variables. To test the differences in CF level among nurses in the three health sectors (three different groups), analysis of variance (ANOVA) test was carried out and the result revealed statistically significant differences (*F* (279, 2) = 8.92, *p* = .000). Based on Scheffe post hoc criterion for multiple group comparison, nurses working in the psychiatric hospital scored significantly higher CF than nurses in governmental hospitals (*P* = .004). Nurses working in educational (training) hospitals scored significantly higher CF than nurses in governmental hospitals (*P* = .003). There was no significant difference in CF between nurses working in psychiatric hospitals compared to educational hospitals (*P* = .764) (Table 3).

**Table 3:** Differences in compassion fatigue level in relation to socio-demographic and substance use

Variable	*M* (SD)	Test value	*P* value
Type of hospital			
Psychiatric hospital	44.4 (19.2)	8.92	.000
Governmental hospital	36.7 (16.5)		
Educational (training) hospital	46.7 (15.1)		
Gender			
Male	44.3 (19.3)	2.43	.015
Female	39.0 (16.4)		
Marital status			
Married	42.8 (17.2)	2.66	.008
Not married	36.9 (18.0)		
Income			
Less or equal 600	40.3 (18.3)	.959	.34
More than 600	42.6 (15.7)		
Smoking			
Yes	44.5 (17.4)	2.19	.029
No	39.4 (17.6)		
Alcohol			
Yes	44.1(21.8)	.912	.363
No	40.6 (17.3)		
Sleeping pills			
Yes	50.5 (19.4)	4.13	.000
No	39.0 (16.7)		
Power drinks			
Yes	47.6 (19.3)	3.10	.002
No	39.4 (16.9)		
Coffee			
Yes	41.3 (18.6)	.493	.62
No	40.1 (15.5)		
Antidepressants			
Yes	48.0 (16.9)	2.82	.005
No	39.7 (17.6)		
Anti-anxiety drugs			
Yes	45.9 (16.9)	2.17	.031
No	39.9 (17.7)		
Amphetamines			
Yes	45.5 (15.3)	1.60	.10
No	40.3 (17.9)		
Analgesic drugs			
Yes	41.2 (20.5)	.257	.797
No	40.7 (15.6)		

Based on socio-demographic variables, and using a Student’s *t* test for comparison for each two independent groups, a significant difference was found in regard to gender (*t* (280) = 2.43, *p* = .015). Male nurses scored higher CF (M = 44.3, SD = 19.3) than female nurses (*M* = 39.0, SD = 16.4). In addition, a statistically significant difference was found among nurses based on marital status (*t* (280) = 2.66, *p* = .008) in which married nurses scored higher CF (*M* = 42.8, SD = 17.2) than the unmarried nurses (*M* = 36.9, SD = 18.0). Compassion fatigue level did not differ significantly (*t* (280) = .959, *p* = .34) in relation to income (Table 3).

In regard to substance use, there was a significant difference in smoking (*t* (280) = 2.19, *p* = .29) as nurses who smoked cigarettes scored higher CF (*M* = 44.5, SD = 17.4) than nurses who did not smoke (*M* = 39.4, SD = 17.6). In regard to sleeping pills, results revealed a significant difference (*t* (280) = 4.13, *p* < .001). Nurses who used sleeping pills scored higher CF (*M* = 50.5, SD = 19.4) than nurses who did not (*M* = 39.0, SD = 16.7). Nurses who used power drinks scored higher CF (*M* = 47.6, SD = 19.3) than nurses who did not (*M* = 39.4, SD = 16.9, *t* (280) = 3.10, *p* = .002). Other significant differences were found in nurses who took antidepressants (*t* (280) = 2.82, *p* = .005) with a mean of (*M* = 48.0, SD = 16.9) compared to nurses who did not (*M* = 39.7, SD = 17.6). Similarly, nurses who took anti-anxiety drugs scored higher CF (*M* = 45.9, SD = 16.9) than nurses who did not (*M* = 39.9, SD = 17.7) with a significant difference (*t* (280) = 2.17, *p* = .031). Finally, results revealed no significant difference in CF scores in regard to coffee use, amphetamines, alcohol or analgesic use (Table 3).

## Discussion

### Compassion fatigue and type of health institution

Work environment and prevailing work conditions, demands, burdens and stressors along with patient’s problems and stories add to the nurses’ potential for CF. Examples of work stressors are paperwork, the electronic medical records, changes in leadership or staffing, accreditation requirements and expectations of best practices [18]. Moreover, nurses often spend considerable time with people who live outside the regular norms of society such as individuals who are extremely poor, victims of abuse or neglect, people with mental illnesses or others who are demented, debilitated, paralytic, comatose or abandoned. Such people with special scenarios absorb the nurses’ physical, intellectual and emotional power; in a way that is adding to the CF provoking effects of work environment which is often stressful, understaffed and overwhelmed with negativity [8, 19].

Those factors indeed play a major role in making some hospitals more CF predisposing environments than others. Hence, our study found that psychiatric health care nurses are more prone to CF than nurses working in non-psychiatric settings. Training-based hospital nurses are more prone to CF than their partners in governmental hospitals, who may be holding less responsibilities and simpler job descriptions. It should not be disregarded here that the mean and the median scores of our sample were within the extremely high risk for CF category, regardless of the hospital type. It is worth noting that that literature comparing CF between different health settings or units are limited in Jordan as well as elsewhere.

### Compassion fatigue and gender

Compassion fatigue differs by some nurse-related socio-demographic variables. In regard to gender, Mooney et al. [20] reported that ICU and oncology male nurses exhibited significantly lower CF (*p* = .014) than female nurses. Those findings were not noted in our study. The variation could be attributed to differences in sample size (86–282), different measurement scales of CF (The Professional Quality of Life (ProQOL) scale to CF self-test 66 items) and different sample characteristics (ICU and oncology nurses in a community hospital versus a more variable mix of different nursing specialities from different types of health settings).

### Compassion fatigue and nurses’ marital status and income level

Nurses’ marital status scored significant differences in relation to CF. Married nurses were more prone to CF than the unmarried. When compared to a research done by Hee and Kyung [21], our finding was inconsistent with theirs. This could be attributed, at least partially, to cultural and economic differences of surveyed populations. Here, in Jordan, the life demands of married people are significantly higher than those who are unmarried; additionally the cost of living is high when compared to personal income [22]. The combination of the escalating burdens of married life, the absence of governmental financial support to families, and the accelerating prices and taxes that touches even the most basic goods, adds extra stress on Jordanian nurses which increase their CF level [23]. It is evident that 74% of the study sample individual income was below 600 Jordanian dinars, and this applies to most Jordanian nurses and regular citizens which is considered low when compared to other Arab countries like Qatar and Kuwait [24].

### Compassion fatigue and substance use

There were significant differences in nurses’ CF scores based on their substance use; namely, cigarette smoking, sleeping pills, power drinks, anti-depressants and anti-anxiety drugs. Nurses who consumed these substances on a frequent basis reported significantly higher CF scores than those who did not; supporting the initial hypothesis. It is recommended that the resiliency characteristics and the coping styles of the participants of similar studies be surveyed in the future.

The term substance use includes prescribed or non-prescribed use, misuse and/or abuse of legal substances such as caffeine, nicotine, alcohol, over-the-counter drugs, prescribed drugs, alcohol concoctions, indigenous plants, solvents, inhalants and illicit drugs. Simply, substance use refers to any substance that, when taken by a person, modifies perception, mood, cognition, behaviour or motor functions, and could be considered a maladaptive behavioural pattern [25].

Substance use among health workers such as nurses is a dangerous behaviour that may lead to negative personal, social and organizational consequences and should be carefully investigated and properly managed [26]. Nurses, like most people with substance use disorders, abuse drugs, tobacco or alcohol to relieve stress and emotional or physical pain. In many cases, the abuse initially helps boost performance; for example when drinking stimulants such as coffee, power drinks or amphetamines, nurses stay wake and energetic during night shifts. This behaviour, if not controlled, will gradually turn into dependence which is an unwanted outcome [27]. The nurses that become abusers sadly become victims, and burdens on the health care system and will eventually require massive interventional rehabilitation programmes to maintain their career lives [28].

When combating stressful feelings of compassion fatigue at work, nurses may turn to negative styles of coping. Maladaptive, non-resilient behaviours of using substances (nicotine, high concentrations of caffeine in the form of power drinks, sleeping pills, antidepressants and anti-anxiety drugs) may be enhanced by the presence of certain negative elements in the work environment. Examples of those elements would be high work demands, low job satisfaction, long duty hours, irregular shifts, fatigue/exhaustion, repetitious duties, periods of inactivity or boredom, irregular supervision and easy access to substances [29].

Nurses working in most health care sectors in Jordan are challenged by multiple negative work environment elements. For example, the ever present nursing shortage, the feminized image of a nurse thereby causing a male-nurse stigma, lower wages when compared to international nursing salaries and poor communication among healthcare team. In addition, Jordanian nurses suffer from a lack of autonomy, non-supportive leadership and/or co-workers, autocratic managers and frequent “floating” of nurses between different units and floors. Besides, nurses often tend to recall the non-supportive or troubled personal relationships, low self-esteem among colleagues, ambiguous nursing roles and the high patient acuities [30–34]. These factors may play a role in pushing nurses toward variable forms of substance use or abuse. Subsequently, it is recommended to study those elements adequately to intervene timely on the behalf of nurses.

### Implications for nursing management

This research strongly indicates that there is a serious nursing compassion fatigue problem which is sometimes accompanied with some sort of substance use behaviour. The fact that the mean and the median scores of CF among nurses was falling within the category of “extremely high risk for CF”, ascertains the need to identify the most risky target groups, like male nurses, married nurses and nurses working in either psychiatric care facilities or overloaded work environments like large training hospitals. There is a need to establish saving managerial strategic interventional plans to rescue those target groups before further personal and organizational damage is inflicted. With regard to the compassion fatigue substance use related behaviour, the health authorities are obliged to follow up this problem comprehensively, specify its prevalence, motives, correlations, outcomes and possible practical solutions. After all healthy nurses will always be vital to an efficient and sustainable Jordanian as well as global healthcare systems.

## Limitations

The sample from this study only included governmental and semi-governmental hospitals, so future studies in Jordan are encouraged to involve both military health services and the private sector and to adopt randomization when picking the included health institutions, type of units and participating subjects in order to enhance the generalizability of the findings. Researchers from other countries and different cultural, social and economic backgrounds are highly recommended to replicate our study to see if the conclusions drawn about nurses’ CF, substance use and the other variables will be the same. Also, comparative studies between different types of health institutions and nursing specialities are required both locally and internationally to set a solid literature data for the phenomena. Besides, future studies are to survey the variables that our study did not cover especially the environmental and personal stressors that lead to compassion fatigue and resultant substance use among nurses as well as the nurses dominating resiliency characteristics and coping styles used when facing diversity and hardship at work.

## Conclusion

This is a pioneer study in Jordan as well as in the region that holds a great value in exploring nurses’ compassion fatigue, associated feelings, characteristics and behaviours. It is necessary to conduct a survey that determines the factors that drive nurses the most towards maladaptive less resilient coping behaviours of substance use; and to further investigate the correlation between CF and such use behaviours.

## Appendix 1: compassion fatigue self-test 66 items

Helping others puts you in direct contact with other people’s lives. As you probably have experienced, your compassion for those you help has both positive and negative aspects. This self -test helps you estimate your compassion status: how much at risk you are of burnout and compassion fatigue and also the degree of satisfaction with you helping others. Consider each of the following characteristics about you and your current situation. Write in the number that honestly reflects how frequently you experienced these characteristics in the last week. Then follow the scoring directions at the end of the self-test.

0 = Never 1 = Rarely 2 = A few times 3 = Somewhat often 4 = Often 5 = Very often

**Table:**

Items about you	
1.	I am happy
2.	I find my life satisfying
3.	I have beliefs that sustain me
4.	I feel estranged from others
5.	I find that I learn new things from those I care for
6.	I force myself to avoid certain thoughts or feelings that remind me of a frightening experience
7.	I find myself avoiding certain activities or situations because they remind me of a frightening experience
8.	I have gaps in my memory about frightening events
9.	I feel connected to others
10.	I feel calm
11.	I believe that I have a good balance between my work and my free time
12.	I have difficulty falling or staying asleep
13.	I have outburst of anger or irritability with little provocation
14.	I am the person I always wanted to be
15.	I startle easily
16.	While working with a victim, I thought about violence against the perpetrator
17.	I am a sensitive person
18.	I have flashbacks connected to those I help
19.	I have good peer support when I need to work through a highly stressful experience
20.	I have had first-hand experience with traumatic events in my adult life
21.	I have had first-hand experience with traumatic events in my childhood
22.	I think that I need to “work through” a traumatic experience in my life
23.	I think that I need more close friends
24.	I think that there is no one to talk with about highly stressful experiences
25.	I have concluded that I work too hard for my own good
26.	Working with those I help brings me a great deal of satisfaction
27.	I feel invigorated after working with those I help
28.	I am frightened of things a person I helped has said or done to me
29.	I experience troubling dreams similar to those I help
30.	I have happy thoughts about those I help and how I could help them
31.	I have experienced intrusive thoughts of times with especially difficult people I helped
32.	I have suddenly and involuntarily recalled a frightening experience while working with a person I helped
33.	I am pre-occupied with more than one person I help
34.	I am losing sleep over a person I help’s traumatic experiences
35.	I have joyful feelings about how I can help the victims I work with
36.	I think that I might have been “infected” by the traumatic stress of those I help
37.	I think that I might be positively “inoculated” by the traumatic stress of those I help
38.	I remind myself to be less concerned about the wellbeing of those I help
39.	I have felt trapped by my work as a helper
40.	I have a sense of hopelessness associated with working with those I help
41.	I have felt “on edge” about various things and I attribute this to working with certain people I help
42.	I wish that I could avoid working with some people I help
43.	Some people I help are particularly enjoyable to work with
44.	I have been in danger working with people I help
45.	I feel that some people I help dislike me personally
Items about being a helper and your helping environment	
46.	I like my work as a helper
47.	I feel like I have the tools and resources that I need to do my work as a helper
48.	I have felt weak, tired, run down as a result of my work as helper
49.	I have felt depressed as a result of my work as a helper
50.	I have thoughts that I am a “success” as a helper
51.	I am unsuccessful at separating helping from personal life
52.	I enjoy my co-workers
53.	I depend on my co-workers to help me when I need it
54.	My co-workers can depend on me for help when they need it
55.	I trust my co-workers
56.	I feel little compassion toward most of my co-workers
57.	I am pleased with how I am able to keep up with helping technology
58.	I feel I am working more for the money/prestige than for personal fulfillment
59.	Although I have to do paperwork that I don’t like, I still have time to work with those I help
60.	I find it difficult separating my personal life from my helper life
61.	I am pleased with how I am able to keep up with helping techniques and protocols
62.	I have a sense of worthlessness/disillusionment/resentment associated with my role as a helper
63.	I have thoughts that I am a “failure” as a helper
64.	I have thoughts that I am not succeeding at achieving my life goals
65.	I have to deal with bureaucratic, unimportant tasks in my work as a helper
66.	I plan to be a helper for a long time
